# Carbohydrate quantity is more closely associated with glycaemic control than weight in pregnant women with type 1 diabetes: Insights from the Diabetes and Pre‐eclampsia Intervention Trial (DAPIT)

**DOI:** 10.1111/jhn.13042

**Published:** 2022-06-14

**Authors:** Alyson J. Hill, C. C. Patterson, I. S. Young, V. A. Holmes, D. R. McCance

**Affiliations:** ^1^ Nutrition Innovation Centre for Food and Health (NICHE), School of Biomedical Sciences University of Ulster Coleraine UK; ^2^ Centre for Public Health Queen's University Belfast Belfast UK; ^3^ Regional Centre for Endocrinology and Diabetes, Royal Victoria Hospital Belfast UK

**Keywords:** body weight, carbohydrate, dietary assessment, pregnancy, Type 1 diabetes

## Abstract

**Background:**

The present study aimed to explore the relationships between carbohydrate intake, body mass index (BMI) and glycaemic control (HbA1c) in pregnant women with type 1 diabetes mellitus (T1DM)

**Methods:**

Secondary analysis of data was undertaken to assess dietary intake in a cohort of women who participated in a randomised controlled trial (RCT) of antioxidant supplementation to prevent preeclampsia (DAPIT^10^). Study‐specific peripheral venous blood samples were obtained for HbA1c at 26 and 34 weeks. Diet was collected using a validated semiquantitative food frequency questionnaire at 26–28 weeks of gestation which assessed dietary intake over 2 weeks. Mean daily average nutrient intakes were analysed using Q Builder nutritional software and SPSS, version 25.

**Results:**

Dietary data were available for 547 pregnant women (72% of cohort) aged 29 years (95% confidence interval [CI] = 28.9–29.9) with average diabetes duration 11.8 years (95% confidence interval = 11.1–12.6). Average body mass index (BMI) (<16 weeks of gestation) was 26.7 kg/m^2^ (95% CI = 26.3 −27, range 18.8–45.6 kg/m^2^); 43% (*n* = 234) were overweight (BMI = 25.0−29.9 kg/m^2^) and 20% (*n* = 112) were obese (BMI ≥ 30 kg/m^2^). Differences in HbA1c and carbohydrate quantity and quality were found when adjusted for age and insulin dose. No differences between BMI group were observed for total carbohydrate and glycaemic control; however, differences were noted in fibre and glycaemic index.

**Conclusions:**

Average quantity of dietary carbohydrate influenced HbA1c when adjusted for insulin dose however, BMI had less impact. More research is required on the relationship between carbohydrate consumption and glycaemic control in pregnancy.

## Key points

- A positive association between glycaemic control (HbA1c) and higher quantity of carbohydrate (>264 g of carbohydrate) consumed in late pregnancy (mean gestational age 27.9 weeks) (*p* = 0.002) was shown in the present study.
- Sixty‐three percent of women were classified as overweight or obese in early pregnancy, and almost 70% gained above the Institute of Medicine recommendations for optimal gestational weight gain. However, no association between body mass index or gestational weight gain and glycaemic control was found.
- This present study suggests that monitoring quantity and type of carbohydrate consumed (and matching insulin doses) may have an impact on glycaemic control and is more closely associated than weight.

## Graphical abstract

Carbohydrate quantity but not body mass index (BMI) is associated with glycaemic control in pregnant women with type 1 diabetes mellitus (T1DM).

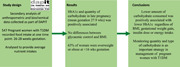

## INTRODUCTION

The prevalence of maternal overweight and obesity is rapidly increasing with 28% and 17%, respectively, reported in the general obstetric UK population.^1^ However, the prevalence is reported to be higher in women with diabetes^2^ although there are limited data available for comparison. One study in Swedish women with type 1 diabetes mellitus (T1DM) reported approximately 50% overweight and obese (35% and 18%, respectively) before pregnancy^3^ and, more recently, a study in a subgroup of CONCEPTT^4^ reported an average body mass index (BMI) of 26.2 kg/m². High maternal prepregnancy BMI in T1DM is strongly associated with elevated risk of adverse pregnancy outcome and, in addition TIDM in combination with overweight and obesity constitutes to a higher risk than either condition alone.^3^ Therefore, BMI is an important risk factor for adverse pregnancy outcomes in T1DM.^3^

Optimising glycaemic control by glucose monitoring, insulin adjustment and appropriate dietary intake is a well‐established goal in pregnancy. Weight gain is a common problem with intensive insulin regimens and has significant metabolic effects outside pregnancy including the development of an atherogenic lipid profile, increased blood pressure and abdominal obesity^5^, ^6^ and cardiovascular risk with long‐term weight retention postpartum.^7^ In addition, excessive gestational weight gain is an independent risk factor for adverse pregnancy outcomes in the general obstetric population^8^ and a major contributor to excessive foetal growth in women with diabetes independent of glycaemic control,^9^, ^10^ with 65% of pregnant women with T1DM reported to have gained more weight than recommended.^11^ Therefore, optimising weight gain during pregnancy reduces the risks of short‐ and long‐term maternal and neonatal outcomes.^8^

Carbohydrate is the main dietary determinant influencing postprandial hyperglycaemia and it is well recognised that the amount, as well as the type, of carbohydrate in meals influences the glycaemic response.^12^ The role of low carbohydrate diets in the management of T1DM is unclear and existing evidence from a recent systematic review was inconclusive at providing guidance for their use in non‐pregnant women with T1DM.^13^ In pregnancy, American Diabetes Association guidance for women with diabetes recommends a moderate carbohydrate diet of 175 g daily to ensure sufficient glucose for both mother and foetus.^8^ The present study aimed to enhance the evidence base in this area by exploring the relationships between BMI, carbohydrate intake and glycaemic control in pregnant women with T1DM.

## METHODS

In total, 547 (72%) pregnant women with T1DM were included in this secondary analysis of data obtained from participants in the Diabetes and Pre‐eclampsia Intervention Trial (DAPIT; *n* = 761 participants) and in whom dietary data were available. DAPIT was a multicentre randomised double‐blind placebo‐controlled trial to investigate the use of antioxidants (vitamins C and E) for prevention of preeclampsia in pregnant women with T1DM.^14^ Details of the methodology have been described previously.^14^, ^15^ In brief, eligible women with T1DM were recruited from 25 joint antenatal‐metabolic clinics in Northern Ireland, Scotland and Northwest England between 2003 and 2008 where they received usual routine care throughout the DAPIT trial. Women were enrolled to the study between 8 and 22 weeks of gestation and where additional study data was collected as outlined in the study protocol.^14^, ^15^ Data were collected at baseline (booking: mean [SD] gestation 8.7 [2.675] weeks and 95% by week 15). Study specific peripheral venous blood samples were obtained for HbA1c at 26 weeks (mean [SD] gestation 26.3 [1.59] weeks) and 34 weeks (mean [SD] gestation 34.2 [1.21] weeks) and stored immediately at −70°C until analysis. HbA1c was measured by spectrophotometry using an automated ILab600 biochemical analyser. As a National Glycohemoglobin Standardisation Programme and International Federation for Clinical Chemistry certified method, the values reported were aligned with the Diabetes Control and Complications Trial system with intra‐ and interassay coefficients coefficients of variation values <2%. Blood glucose profiles were recorded by participants and provided to researchers from study diary readings. Early pregnancy BMI (kg/m^2^) was calculated from a measured weight recorded <16 weeks of gestation. Women were categorised using the World Health Organization classification: underweight (BMI ≤ 18.5 kg/m^2^); normal weight (BMI = 18.5–24.99 kg/m^2^); overweight (BMI = 25.0–29.99 kg/m^2^); obese class I (BMI = 30–34.99 kg/m^2^): obese class II (BMI = 35.0–39.99 kg/m^2^): obese class III (BMI ≥ 40 kg/m^2^). Gestational weight gain was then calculated only for participants who had accurate measured weights at all time‐points (*n* = 249). This was achieved by calculating the total amount of weight gained (from week 13 to last weight measurement) and dividing by the number of weeks of gestation to give kg/week^1^. Informed consent was given by all subjects who participated in the present study and ethical approval was obtained and the West Midlands Multicentre Ethics Research Committee provided ethical approval for DAPIT^14^ (MREC 02/7/016).

### Dietary evaluation

Pregnant women completed a validated semiquantitative food frequency questionnaire (FFQ) between 26 and 28 weeks of gestation (mean [SD] gestation 27.9 [1.56] weeks). The FFQ included 72 quantitative and qualitative questions of which 48 focused on frequency of consumption of cereals, meats, poultry and fish, fats and oils, sweet foods, fruits and vegetables and drinks. Researchers trained by a specialist diabetic dietitian instructed women to record their usual intake of foods consumed close to 26 weeks of gestation over a 2‐week period using a standardised protocol. Questions were also asked regarding frequency of meals and supplements taken. Portion sizes when not specified in the FFQ were estimated based on standard UK portion sizes. Dietary questionnaires were returned to the study dietitian for checking, assessment and analysis. Mean average nutrient intakes of each participant were calculated from the FFQ using the nutritional software package Q‐Builder (Questionnaire Design System, version 2.0; Tinuviel Software) where the frequency of consumption of foods was converted into foods and weights which generated a mean average daily nutrient intake. Estimated energy intakes calculated from the FFQ were validated against those obtained from a 7‐day weighed food record (*n* = 68), which showed significant positive correlations for nutrients (range for nutrients *r* = 0.14–0.72; Bronte unpublished 2012). Under‐reporting of energy intake (EI) was determined by calculating basal metabolic rate (BMR) using published equations based on age, prepregnancy weight and height.^16^ Using the Goldberg method, the levels of under‐reporting were predicted, using the ratio of energy intake (EI reported) to estimated BMR (BMR estimated).^17^ A ratio of ≤ 1.2 may indicate under‐reporting and a ratio of ≤ 0.9 is a sign of definite under‐reporting.^17^ Subjects were divided into three groups ‘definite under‐reporters’ if EI:BMR ratio was ≤ 0.9; ‘potential under‐reporters’ if ratio was > 0.9 to ≤ 1.2 and ‘normal reporters’ > 1.2.^18^ Analysis was run both with and without definite under‐reporters.

### Statistical analysis

Differences between groups were assessed using analysis of covariance, adjusted for age, on log‐transformed data where appropriate. Geometric means were back transformed from natural logs. Associations between nutrient intake and glycaemic control were analysed using Pearson's correlation coefficients (*r*) on log‐transformed data and adjusted for age, BMI and insulin/kg^1^/day^1^. Determinants of poor glycaemic control were identified using binary logistic regression. *p* < 0.05 was considered statistically significant. A test for tend in means was carried out. All data was analysed using SPSS, version 25.0 (IBM Corp.).

## RESULTS

Table 1 shows maternal characteristics of participants (*n* = 547 72%) included in this secondary analysis, according to BMI category. No significant differences were found between those participants who were included (dietary information available) in the analysis: (mean [SD] [*n* = 547] with BMI = 27.5 [4.6] kg/m^2^) and those excluded (no dietary information available) (*n* = 214) with BMI = 27.2 (4.8) kg/m² (*p* = 0.49). Subjects were almost exclusively Caucasian (98%) with a mean age of 29.4 years (95% confidence interval [CI] = 28.9–29.9) and average diabetes duration of 11.8 years (95% CI = 11.1–12.6). At booking, mean (SD) gestation was 8.71 (2.675) weeks, mean HbA1c (International Federation of Clinical Chemistry and Laboratory Medicine [IFCC]) was 59.6 mmol/mol^1^ (95% CI = 58.4–60.8) (mean HbA1c = 7.7%; 95% CI = 7.5–7.8) and average BMI was 26.7 kg/m^2^ (95% CI = 26.3−27.00, range 18.8–45.6 kg/m^2^); 0% of patients were classed as underweight, 37% (*n* = 201) were classed as normal weight, 43% (*n* = 234) were classed as overweight and 20% (*n* = 112) were classed as obese. Of those categorised as obese, 14% (*n* = 75) were classed as obese Class I, 5% (*n* = 29) were classed as obese Class II and 1% (*n* = 8) were classed as obese Class III.

**Table 1:** Maternal characteristics of pregnant women with type 1 diabetes mellitus by prepregnancy body mass index (BMI)

	BMI					
		All	Healthy	Overweight	Obese	*p* value
			(18.5–24.99 kg/m^2^)	(25.0–29.99 kg/m^2^)	(30.0+ kg/m^2^)	
			*n* = 201	*n* = 234	*n* = 112	
	*N*	Mean (95% CI)	Mean (95% CI)	Mean (95% CI)	Mean (95% CI)	
Age at booking (years)	547	29.4 (28.9–29.9)	28.6 (27.8–29.4)^a^	29.5 (28.8–30.2)^ab^	30.7 (29.7–31.8)^b^	0.005*
Duration of diabetes (years)	547	11.8 (11.1–12.6)	10.8 (9.6–12.1)	12.9 (11.8–14.0)	11.6 (10.0–13.4)	0.082
Weight (<16 weeks) (kg)	547	71.5 (70.5–72.5)	61.8 (61.0–62.6)^a^	73.0 (72.2–73.8)^b^	88.7 (86.4–91.0)^c^	<0.0001*
Height (m)	547	163.7 (163.1–164.3)	164.8 (163.8–165.7)^a^	163.6 (162.8–164.4)^ab^	161.8 (160.3–163.4)^b^	0.001*
BMI (<16 weeks) (kg/m^2^)	534	26.7 (26.3–27.0)	22.8 (22.5–23.0)^a^	27.3 (27.1–27.5)^b^	33.9 (33.3–34.5)^c^	<.0001*
HbA1c (<13 weeks) (%)	531	7.7 (7.5–7.8)	7.7 (7.5–7.9)	7.7 (7.6–7.9)	7.4 (7.2–7.6)	0.171
IFCC (<13 weeks) (mmol/mol^1^)	531	59.6 (58.4–60.8)	59.9 (57.7–62.1)	60.6 (58.8–62.5)	57.1 (54.8–59.5)	0.178
Insulin (26 weeks) (units/kg^1^/day^1^)	461	0.91 (0.88–0.93)	0.86 (0.82–0.91)	0.92 (0.88–0.96)	0.94 (0.87–1.01)	0.059
Gestation at delivery (weeks)	547	36.7 (36.5–36.9)	36.6 (36.3–36.9)	36.8 (36.5–37.0)	36.6 (36.2–37.0)	0.774
Weight gain (kg/week^1^)	249	0.50 (0.47–0.53)	0.52 (0.47–0.57)^ab^	0.51 (0.47–0.55)^a^	0.41 (0.32–0.51)^b^	0.040*
Cholesterol (mmol/L^1^)	502	4.82 (4.74–4.90)	4.68 (4.56–4.81)	4.91 (4.79–5.03)	4.91 (4.71–5.11)	0.059
Vitamin C (mmol/L^1^)	483	38.8 (36.5–41.2)	40.4 (36.7–44.5)	39.7 (36.0–43.8)	34.0 (30.1–38.3)	0.114

*Notes*: Blood cholesterol and vitamin C randomisation visit; mean (SD) 14 (3.36) weeks of gestation. Gestational weight gain was calculated only for participants with accurate weights at all time‐points (*n* = 249). CI, confidence interval.

^a,b,c^Mean values within a row with different superscript letters indicate a significant difference between groups.

* Significant difference between variable mean between BMI category by ANCOVA analysis with adjustment for age (*p* < 0.05).

Women who were obese were significantly older (*p* < 0.005) compared to normal weight women, although no differences were observed between duration of diabetes, glycaemic control and units of insulin/kg^1^/day^1^ at booking. No women were receiving insulin using an insulin pump at the time of the study. Obese women gained significantly less weight during pregnancy (0.41 kg/week^1^) compared to healthy weight women (0.52 kg/week^1^) (*p* < 0.040).

Dietary data were available for 72% (*n* = 547) of participants in the DAPIT^14^ study at mean (SD) gestational age of 27.9 (1.56) weeks, and no differences in BMI were observed between those women who completed dietary assessment (BMI 27.5 [4.6] kg/m^2^) (*n* = 547) and those who did not (BMI 27.2 [4.8] kg/m²) (*n* = 214) (*p* = 0.49).

Average daily energy intake was 6.892 MJ (95% CI = 6.737–7.050) for the group as a whole (Table 2). Daily average intakes for most nutrients did not differ between BMI categories, although exceptions were fibre, where the overweight (15.7 g) and obese (15.5 g) groups had significantly lower intakes than normal weight women (17 g) (*p* = 0.013). Overall, the diets of the cohort comprised, on average, approximately 55% carbohydrate, 30% fat and 18% protein of total energy, although no differences were observed between BMI groups (Table 2). No differences across the three BMI categories of normal weight, overweight or obese were observed for blood glucose profile at 26 weeks (Table 3) and likewise at all visits.

**Table 2:** Nutrient intake of cohort at an average 26 weeks of gestation split by prepregnancy body mass index (BMI) categories

	BMI				
	All (*n* = 547)	Healthy (18.5–24.99 kg/m^2^)	Overweight (25.0–29.99 kg/m^2^)	Obese (30.0+ kg/m^2^)	*p* value
		(*n* = 201)	(*n* = 234)	(*n* = 112)	
Energy (kJ)	6892 (6737–7050)	7064 (6788–7351)	6815 (6598–7040)	6751 (6409–7112)	0.377
Carbohydrate (g)	225 (220–230)	226 (219–236)	224 (217–231)	221 (210–232)	0.787
Sugars total (g)	79.3 (76.9–81.7)	79.3 (75.3–83.5)	80.3 (76.8–84.0)	77.1 (71.7–82.8)	0.563
% Energy CHO	55.1 (54.6–55.6)	54.6 (53.8–55.5)	55.4 (54.6–56.2)	55.2 (54.0–56.3)	0.398
% Energy fat	29.5 (29.0–30.0)	30.1 (29.3–30.9)	29.0 (28.2–29.8)	29.6 (28.6–30.8)	0.201
% Energy protein	17.9 (17.7–18.2)	17.9 (17.5–18.3)	18.1 (17.7–18.5)	17.7 (17.1–18.2)	0.343
Fibre Englyst (g)	16.1 (15.6–16.6)	17.0 (16.2–17.8)^a^	15.7 (15.0–16.4)^b^	15.5 (14.4–16.7)^b^	0.013*
Iron (mg)	10.7 (10.5–11.0)	11.4 (10.9–11.9)^a^	10.4 (10.1–10.8)^b^	10.4 (9.8–11.0)^b^	0.002*
Vitamin D (µg)	2.4 (2.3–2.6)	2.7 (2.5–3.0)^a^	2.2 (2.0–2.5)^b^	2.2 (2.0–2.5)^b^	0.003*
Vitamin C (mg)	108 (103–113)	109 (101–118)	109 (101–117)	106 (95–118)	0.727
Glycaemic load	124 (121–127)	126 (121–131)	122 (118–127)	122 (116–129)	0.671
Glycaemic index	55.2 ± 3.4	55.5 ± 3.1^a^	54.7 ± 3.5^b^	55.4 ± 3.4^ab^	0.029*
EI:BMR ratio	0.82 (0.80–0.84)	0.88 (0.85–0.92)^a^	0.80 (0.78–0.83)^b^	0.75 (0.71–0.79)^b^	< 0.0001*

^a,b^Mean values within a row with different superscript letters indicate a significant difference between groups.

Abbreviations: BMR, basal metabolic rate; CHO, carbohyrdrate; EI, energy intake.

* Significant difference between variable mean between BMI category by analysis of covariance, adjusted for age (*p* < 0.05).

**Table 3:** Blood glucose profile at 26 weeks of gestation by body mass index (BMI) categories

	BMI				
	*N*	Healthy	Overweight	Obese	*p* value
		(18.5–24.99 kg/m^2^)	(25.0–29.99 kg/m^2^)	(30+ kg/m^2^)	
Fasting glucose (mmol/L)	481	5.8 (5.5–6.1)	5.8 (5.5–6.1)	5.9 (5.5–6.4)	0.886
Glucose 1 h postbreakfast (mmol/L)	358	7.1 (6.5–7.7)	7.3 (6.8–7.8)	7.2 (6.5–8.0)	0.886
Glucose prelunch (mmol/L)	476	5.6 (5.3–6.0)	5.8 (5.5–6.2)	5.9 (5.4–6.4)	0.511
Glucose 1 h postlunch (mmol/L)	346	6.8 (6.2–7.4)	6.6 (6.2–7.1)	6.7 (6.0–7.4)	0.901
Glucose predinner (mmol/L)	470	5.8 (5.5–6.2)	5.7 (5.4–6.0)	5.6 (5.2–6.1)	0.947
Glucose 1 h postdinner (mmol/L)	340	6.7 (6.1–7.2)	7.2 (6.7–7.7)	6.7 (6.2–7.3)	0.253
Glucose presupper (mmol/L)	412	6.1 (5.7–6.5)	6.2 (5.8–6.6)	5.8 (5.3–6.3)	0.458
Glucose 1 h postsupper (mmol/L)	219	7.0 (6.4–7.6)	6.7 (6.1–7.4)	6.9 (6.1–7.7)	0.731

* Significant difference between variable mean between BMI category by analysis of covariance, adjusted for age (*p* < 0.05).

Under reporting of energy intake was observed (EI:BMR) in the overweight and obese groups with significantly greater under reporting in both groups (*p* < 0.0001) as shown in Table 2. When the ‘definite’ under‐reporters (EI:BMR < 0.9) (20.3% of sample) were excluded from the analysis, mean energy intake increased to 7.158 MJ; however, no significant differences between BMI groups were observed for energy and macronutrient intake.

No relationship was seen between average nutrient intake which was assessed at 26–28 weeks of gestation and glycaemic control (HbA1c mmol/mol^1^) at that time. However, differences were observed between energy (kJ) (*p* < 0.041), carbohydrate (g) (*p* < 0.023), fibre (g) (0.047) and glycaemic load (*p* < 0.016) when adjusted for age, BMI and insulin dose (insulin/kg) (Table 4). Linear regression showed that total carbohydrate intake, energy and glycaemic load were strongly correlated (*r* > 0.8); energy and glycaemic load were therefore omitted from the regression model. Carbohydrate was the strongest predictor of glycaemic control (>48 mmol/mol^1^) at this time with the highest quintile of carbohydrate (>264 g/day^1^) being the strongest predictor (*p* = 0.002) of higher HbA1c (Table 4). Duration of diabetes was a significant determinant (*p* = 0.012); however, BMI and age were not found to be significant determinants.

**Table 4:** Determinants of poor glycaemic control (HbA1c IFCC 48 mmol/mol^1^ ≥ 6.5%)

		OR	95% CI	*p*
Age		0.419	(0.132–1.331)	0.140
Duration (years)		0.688	(0.514–.921)	0.012*
BMI	Healthy (18.5–24.99 kg/m^2^)	Ref	–	–
	Overweight (25.0–29.99 kg/m^2^)	1.213	(0.758–1.942)	0.421
	Obese (30.0+ kg/m^2^)	1.076	(0.615–1.885)	0.797
Carbohydrate intake	< 191.3 g	Ref	–	–
	191.4–225.1 g	2.194	(1.188–4.052)	0.012*
	225.2–264.0 g	2.022	(1.130–3.620)	0.018*
	> 264.1 g	2.510	(1.385–4.547)	0.002*

Abbreviations: BMI, body mass index; CI, confidence interval; OR, odds ratio.

* Binary logistic regression (*p* < 0.05). Patients were categorised into quartiles according to their carbohydrate intake.

## DISCUSSION

In the present study, a secondary analysis of data collected as part of the DAPIT examined the relationships between glycaemic control, BMI and carbohydrate intakes in pregnant women with T1DM, which showed a positive association between HbA1c and quantity of carbohydrate consumed in late pregnancy (mean [SD] gestational age of 27.9 [1.56] weeks), but no relationship was observed between glycaemic control and BMI.

Positive associations between the quantity of carbohydrate intake and glycaemic control have been previously reported in both pregnant and non‐pregnant women with type 1 diabetes.^19^ The present current study showed that the quantity of carbohydrate consumed in late pregnancy (approximately 26–28 weeks) showed a positive association between HbA1c and that a higher quantity of carbohydrate (> 264 g of carbohydrate) was positively associated with HbA1c (*p* = 0.002). However, because dietary intake was assessed at only one time point, no conclusion can be drawn about other stages of pregnancy, although a previous study in early pregnancy (64 days) showed that a lower amount of carbohydrate was associated with better glycaemic control.^19^ However, it must be noted that the American Diabetes Association recommends a minimum intake of 175 g of carbohydrate daily.^8^

Approximately 96% of women in the present study reported an average carbohydrate intake > 175 g/day^1^, which is recommended to provide sufficient supplementation of glucose to the mother and foetus for fetal growth and brain development.^8^ Average carbohydrate intakes observed in the present study were 55% of total energy (range 35–73%) with an average intake of 225 g/day^1^ (95% CI = 220–230 g/day^1^, which is classed as ‘high’ carbohydrate (> 55% total energy intake) ^13^, ^20^ and is similar to the 56% reported in a subgroup of CONCEPPT trial^4^ and also a review^21^ that reported the range 45%–64%; therefore, evidence on the minimum amount of carbohydrate that is safe in the management of T1DM and also the type of carbohydrate and the relationship with glycaemic control and pregnancy outcome is required.^21^

Carbohydrate counting is an integral component of modern dietary management; however, at the time of the present study, women were not routinely educated on insulin adjustment. All women were on multiple daily injections with less flexibility, although all had good awareness of carbohydrate and the effect on glycaemia. Evidence from one study showed that women using carbohydrate counting had lower HbA1c,^19^ and it is now recommended that carbohydrate counting and insulin dose adjusting is an effective strategy for optimising blood glucose control,^12^ although no optimal amount of carbohydrate is suggested.^12^

In addition to the quantity of carbohydrate, the quality has also been shown to be an important determinant of postprandial glycaemic response.^22^ Small positive differences in glycaemic control were shown in the present study between fibre intake and glycaemic load when adjusted for age, BMI and insulin dose. Average fibre intake was 16 g/day^1^, which is below the recommended (12–24 g/day^1^ Englyst method); therefore, those women with T1DM should be encouraged to achieve the UK recommended (30 g AOAC method/23–24 g Englyst) because evidence suggests that diets high in fibre may be beneficial for those with T1DM.^12^

It is well known that obesity is associated with increased insulin resistance and decreased insulin sensitivity^23^ and women may therefore have poorer control in early pregnancy than women with normal or slightly elevated BMI.^3^ In the present study, 63% (*n* = 346) of women were overweight or obese in early pregnancy (<16 weeks of gestation); however, no relationships between BMI and glycaemic control were noted from measurements recorded routinely before pregnancy (HbA1c 6 months prior to pregnancy, *p* = 0.934) or in early pregnancy (HbA1c at booking, *p* = 0.171) were shown. Likewise, no significant differences in glycaemic control at 26 weeks of gestation across the normal, overweight or obese groups in terms of fasting glucose, 1 h postprandial or premeal glucose levels were found.

The present study shows a higher prevalence of overweight and obesity in early pregnancy than that found in a study by Persson *et al*.,^3^ which reported 53% in pregnant women with type 1 diabetes compared to reported rates of 45%^1^ and 41%^24^ in the general obstetric non‐diabetic population. It is recommended that pregnant women with diabetes who have a prepregnancy BMI of > 27 kg/m² be given weight reduction advice prior to pregnancy.^12^ However in the DAPIT study, over one‐third of women (39%) considered their pregnancy unplanned^25^ and, additionally, the proportion of women who reported receiving prepregnancy counselling was significantly lower among those with unplanned pregnancies.^24^ Therefore, although there was no relationship found between unplanned pregnancy and BMI in the present study, it is nevertheless recommended that all women with T1DM receive advice about pregnancy planning^26^ and this should also include weight management dietary advice.^12^

Associations between obesity in T1DM women and aggravated insulin resistance leading to the requirement for increased insulin doses to maintain optimal glycemic control have been reported^11^ and it is recognised that weight gain with intensive insulin regimens increase BMI by 5 kg/m^2^ in nonpregnant women.^27^ However, the present study did not show any association between dose of insulin (unit/kg^1^/day^1^), BMI or gestational weight gain and glycaemic control. Almost 70% (of 249 women with data available) gained above the recommendations for optimal gestational weight gain relative to prepregnancy BMI,^8^ which is higher than that in a similar study of T1DM that reported 54%^10^ (of 115 women). In the present study, obese women were shown to gain significantly less weight (0.41 kg/week^1^) than normal weight women (0.52 kg/week^1^) (*p* = 0.040) between 13 and 36 weeks of gestation, which is consistent with another study^28^ reporting that weight gain decreased with increasing prepregnancy BMI. Given that improved glycaemic control is required to reduce the risk of adverse pregnancy outcomes and that weight management plays a central role in achieving optimal control, a combination of lifestyle interventions and modern insulin treatments and adjustment that are associated with less weight gain should be promoted. HbA1c does not reliably reflect changes in mean blood glucose in pregnancy; however, higher levels may still be useful as a marker of poor glycaemic control.

No difference in dietary energy intake was observed in relation to glycaemic control, regardless of BMI. Average daily energy intake was 6.892 MJ (95% CI = 6.737–7.050) for the group as a whole, although, when under‐reporters (20%) were removed, this increased to 7.158 ± 1.879 MJ, which is broadly similar to those in a general obstetric population (non‐diabetic), such as in Sheffield^29^ (7.8 MJ), Bristol^30^ (7.7 MJ) and Ireland^18^ (8.0 MJ), but slightly higher than in a similar study in pregnant women with T1DM (6.99 MJ).^4^

The present study has a number of strengths. This secondary analysis of data collected from women with type 1 diabetes who participated in the DAPIT^14^ study characterise nutrient intakes at the same time as including accurate BMI measurements and gestational weight gain in a large cohort of UK pregnant women (*n* = 547) exclusively with T1DM. The present study used BMI data on women who had an early BMI weight <16 weeks of gestation to ensure consistency and comparability. This is unlike other studies in pregnant women that have used recalled antenatal weight measurements, which has the potential for bias by misclassifying women. The present study relied on self‐reported dietary data, which was collected using a validated semiquantitative FFQ at one time point (26–28 weeks of gestation), using estimated portion sizes; therefore, as with all dietary surveys, recording food intake has limitations and may limit generalisation of results. Likewise, the results may not be truly representative of pregnant women with T1DM in the UK because participants were recruited to an RCT study and only women who correctly completed food records were included.

In conclusion, the present study showed that a lower amount of carbohydrate consumed was positively associated with lower HbA1c regardless of BMI, gestational weight gain, insulin dose or energy intake. Therefore, the present study suggests that monitoring quantity and type of carbohydrate consumed (and matching insulin doses) may have an impact on glycaemic control and be an important strategy for optimising glycaemic control. No association between BMI or gestational weight gain and glycaemic control was found; however, 63% of women were overweight and obese in early pregnancy and almost 70% gained above the recommendations for optimal gestational weight gain. Accordingly, routine monitoring of weight at set time points during pregnancy may be justified.

## AUTHOR CONTRIBUTIONS

Alison J. Hill wrote the manuscript. D. R. McCance and I. S. Young designed and promoted the study. I. S. Young sought ethics approval. V. A. Holmes coordinated all aspects of the trial and managed the data. C. C. Patterson provided statistical advice and analysed the data. All authors helped to prepare the final report.

## CONFLICTS OF INTEREST

AJH has received speaker honoraria. DRM and ISY have received speaker honoraria and have participated in advisory boards. CCP and VH declare that they have no conflicts of interest.

## ETHICS STATEMENT

DAPIT study was given a favourable ethical opinion by West Midlands Multicentre Ethics Research Committee (MREC 02/7/016).

### PEER REVIEW

The peer review history for this article is available at https://publons.com/publon/10.1111/jhn.13042

## TRANSPARENCY DECLARATION

The lead author affirms that this manuscript is an honest, accurate and transparent account of the study being reported (the author will delete as appropriate). The lead author affirms that no important aspects of the study have been omitted and that any discrepancies from the study as planned have been explained.
